# Liquid biofertilizers as a sustainable solution for agriculture

**DOI:** 10.1016/j.heliyon.2022.e12609

**Published:** 2022-12-23

**Authors:** Mintallah Mousa A. Allouzi, Safa Mousa A. Allouzi, Zi Xiang Keng, Christina Vimala Supramaniam, Ajit Singh, Siewhui Chong

**Affiliations:** a Department of Chemical and Environmental Engineering, Faculty of Science and Engineering, University of Nottingham, Broga Road, 43500 Selangor, Malaysia; b Department of Medicine, Faculty of Medicine, Bioscience, and Nursing, MAHSA University, Jalan SP 2, Bandar Saujana Putra, 42610 Jenjarom, Selangor, Malaysia; c School of Biosciences, Faculty of Science and Engineering, University of Nottingham, Broga Road, 43500 Selangor, Malaysia

**Keywords:** Biofertilizer, Green fertilizer, Liquid fertilizer, Plant growth, Soil enhancement

## Abstract

This paper provides a mini review of liquid biofertilizers, which have been proven to perform better than the other forms in lasting for longer periods of time, improving crop quality, and requiring less amounts for application. The production of liquid biofertilizers, types of liquid inoculants, and their effect on plant growth are covered in this review. Liquid biofertilizers can be made from wastes and by-products of several industries, making zero or near-zero discharge possible and thus gearing towards circular economy. Despite their usefulness in enhancing crop quality and eco-friendliness, in order to compete with chemical fertilizers, there are a number of challenges to overcome, such as extending the shelf life, making them more susceptible to seasonal climate conditions and soil types, and development of suitable machineries for production and application. More field trials, cost-benefit analysis and long-term studies should also be evaluated for commercialization purposes.

## Highlights

• This paper presents state-of-the-art review of liquid biofertilizers.
• Liquid biofertilizers were compared and their production processes were outlined.
• Sources of liquid inoculants were reviewed with plant growth analysis compared.
• Shelf life, machinery and susceptibility to climate and soil hinder its widespread.
• More field trials, cost analysis, and multistakeholder partnerships are essential.

## 1 Introduction

The current increase in the world population to 7.8 billion has placed an increasing demand on agricultural crops, thus posing great challenges in terms of how to feed such a large population (Worldometers, 2021). According to the United Nations Food and Agriculture Organization’s estimates, the demand on agricultural crops will increase to 60% by the year 2030 (Bruinsma, 2002). To reach self-sufficiency, chemical fertilizers have been widely used by countries to increase crop yield. However, these chemical fertilizers are causing serious environmental pollution by reducing water-holding capacity in the soil and thus its fertility, increasing soil acidity, and reducing the number of microorganisms, resulting in nutritional imbalances in the soil (Nosheen et al., 2021). In addition, these hazardous substances are not taken up by plants, but accumulate in ground water affecting the soil negatively. Therefore, it is vital to shift the focus to the production of safe and environmentally friendly methods for sustainable crop production.

Beneficial microorganisms present in the soil are often utilized for sustainable crop production as they have long lasting effects on the soil fertility (Mitter et al., 2021; Umesha et al., 2018). Knowledge of the use of microorganisms to increase soil fertility began as early as 1901, when the American chemist, Harvey W. Wiley, delivered a speech to the Franklin Institute in which he spoke about the possibility of using microorganisms (*Rhizobium* sp.) to enhance soil fertility. Discovering that organisms capture nitrogen laid the groundwork for further developments in scientific research related to agriculture. He described “Alinite and Nitragin”, a commercial product for fixing nitrogen in the soil, but these products needed more development by multiplication of the organisms in favorable environments (Knight, 1878).

Biofertilizers consist of single or multiple strains of microbes like algae, bacteria, and fungi that enhance the plant growth through colonization of the rhizosphere and the interior of the plant, and through enhanced nutrient availability that can be added to the seeds, plant surface, or the soil (Nosheen et al., 2021; Riaz et al., 2020). Biofertilizers are not only responsible for enhancing soil physiochemical properties, but also affecting the structure and function of microorganisms via changes in the microbial carbon, microbial diversity, and the community level physiological profiling (Aponte et al., 2022; Javoreková et al., 2015). Even so, not all native soil microbes interact the same with biofertilizers, since the addition of plant growth promoting rhizobacteria (PGPR) in the rhizosphere may enhance certain microbial groups or inhibit them, and in some cases PGPR does not affect the native microbial population at all (Mącik et al., 2020).

Consumer awareness about the hazards of chemical fertilizers, soil deterioration, and nitrate emissions, as well as government measures, are increasing with years. Hence, the market for biofertilizers is expected to increase from 2.3 billion USD in 2020 to 3.9 billion in 2025 (Mehra, 2020). Biofertilizers are known for their ability to provide plants with nutrients such as nitrogen, phosphate, zinc, phosphorus and also help in promoting plant growth (Szilagyi-Zecchin et al., 2016). Since carrier based biofertilizers have a short shelf life, low cell count, and difficulties in storage and handling, liquid biofertilizers which a have a high cell count of more than 10^9^ were developed to overcome these problems (Nagarajan, 2021).

Production of low cost, effective biofertilizers involves multiple phases, starting from choosing the suitable carrier, isolation and screening of microbes to find the most potent one, to undergoing several tests, before scaling it up from flask-stage to commercial stage (Stamenković et al., 2018; Vassileva et al., 2021). It is also important to find cheaper raw materials that are high in nutrients, carbon, and nitrogen source and use it as a substrate or possible liquid media to culture microorganisms. Some industries are required to pay to get rid of their waste or have a difficult time in treating their wastes. Thus, these wastes and byproducts can be used as possible substrates to develop a sustainable, eco-friendly biofertilizer (He et al., 2020; Namfon et al., 2017). Moreover, biofertilizers can be tailored to provide plants with nitrogen, phosphate, zinc, or other nutrients in different soil types using certain types of bacteria compared to using none or chemical fertilizers (He et al., 2019).

In the light of these features, this review first compares biofertilizers with chemical and organic fertilizers, then evaluates the production processes and compares the liquid inoculants and their impacts on plant growth. The development of liquid biofertilizers is discussed based on how it is developed from different substrates, or by-products and wastes of some industries. Finally, the challenges and recommendations for future studies are discussed to improve the production and development of liquid biofertilizer, benefiting its expansion and commercialization for the agricultural industries.

## 2 Biofertilizers

### 2.1 Biofertilizers in general

Biofertilizers are one of the most promising ways to increase crop production while staying environmentally friendly (Kour et al., 2020). Unlike organic fertilizers which consist of animal manure, compost, slurry waste, peat, bones, and blood meal (Hazra, 2016; Raimi et al., 2017), biofertilizers contain one or more living microorganisms (i.e., bacteria, fungi, algae) alone or in combination that settle down in the rhizosphere and enhance soil productivity by fixing down atmospheric nitrogen and solubilizing different nutrients, thereby exerting direct or indirect beneficial effects on crop growth and yield through different mechanisms (El-Ramady et al., 2018; Mącik et al., 2020). In organic fertilizers, some organisms like earthworms need to convert the fertilizer into useful material which plants can absorb easily. Plant-growth promoting rhizobacteria is the most used bacteria in producing biofertilizers since it enhances plant growth by releasing potassium (K), fixing nitrogen (N), solving phosphorus (P), and producing hormones (Lu et al., 2020). Biofertilizers come in solid, liquid, polymer entrapped formulations, and fluidized bed dry formulations (Mącik et al., 2020). Table 1 shows the differences between biofertilizers, chemical, and organic fertilizers with the pros and cons analyzed.

**Table 1:** Comparison between biofertilizers, chemical and organic fertilizers.

	Biofertilizer	Chemical fertilizer	Organic fertilizer
Definition	Contains microorganisms that apply direct or indirect benefits to the plant growth through various mechanisms	Consists of chemical composition of various elements and minerals needed to enhance plant growth and produced from synthetic materials	Comes from the decomposition of organic waste naturally and is either from plant sources (green manure) or animal sources (animal manure).
Forms	Solid, liquid, polymer entrapped, and fluidized bed dry formulations	Pellets, tablets, granules, liquid, and spikes	Meal, powder. pellets, slurry waste, worm castings, and compost
Example (Barker, 2019; Carpanez et al., 2022; Ma et al., 2021)	1. N-fixing 2. P-solubilizing 3. P-mobilizing 4. Biofertilizer for micronutrients 5. Biofertilizer for beneficial nutrients 6. Plant-growth promoting rhizobacteria 7. Silicate solubilizing 8. Sulfur oxidizing 9. Zinc solubilizing 10. Potassium solving 11. Algae	1. Granular triple super phosphate 2. Potassium chloride 3. Urea 4. Anhydrous ammonia 5. Mono potassium phosphate 6. Ammonium nitrate 7. Ammonium phosphate 8. Ammonium sulphate 9. Calcium nitrate	1. Blood meal: nitrogen fertilizer 2. Bonemeal: source of nutrients 3. Biochar 4. Sewage sludge 5. Soy bean meal 6. Rapeseed meal 7. Bio digestates 8. Sugar cane vinasse
Advantages	1. Increases soil quality and fertility 2. Improves root proliferation 3. Harnesses N_2_ from the atmosphere 4. Microorganisms break down complex organic materials into simpler ones 5. Renewable source of plant nutrients 6. Water stress resistance 7. Reduces plant diseases and pests 8. Increases crop yield as well as weight both in green houses or in fields 9. Reduces the dose required for cultivated crops	1. Readily available 2. Easy to use 3. Different formulas 4. Fast acting 5. Cheap	1. Nutrient release 2. Increases grass and plant resistant to diseases and insect attacks 3. Low salt concentration (does not burn plants) 4. Promotes a healthy soil ecosystem 5. Promotes soil productivity
Disadvantages	1. Low nutrient density 2. Needs different machinery to apply than that used for chemical fertilizer 3. Hard to locate in certain areas 4. Needs large amounts for crops 5. The effectiveness of biofertilizer depends on the surrounding agro-environment 6. Interactions between biofertilizer and soil microbes may inhibit plant growth, immobilize plant nutrients, and produce phytotoxic substances 7. Slower effect on plant growth 8. Prolonged use will inevitably deplete in phosphorus	1. Likely to damages the environment 2. Affects human health 3. Less likely to enhance soil quality 4. Increases soil acidity 5. Loss of bacteria 6. Atmospheric pollution 7. Soluble in water	1. Depends on soil’s microorganisms for nutrients release 2. Hard application of some forms 8. Can attract bugs and pets 9. Limited availability in some areas 10. May contain pesticide residue
Nutrients	Not a source of nutrients, but helps the plants to access them from the rhizospheric region	A source of nutrients	A source of nutrients

**Figure 1:**
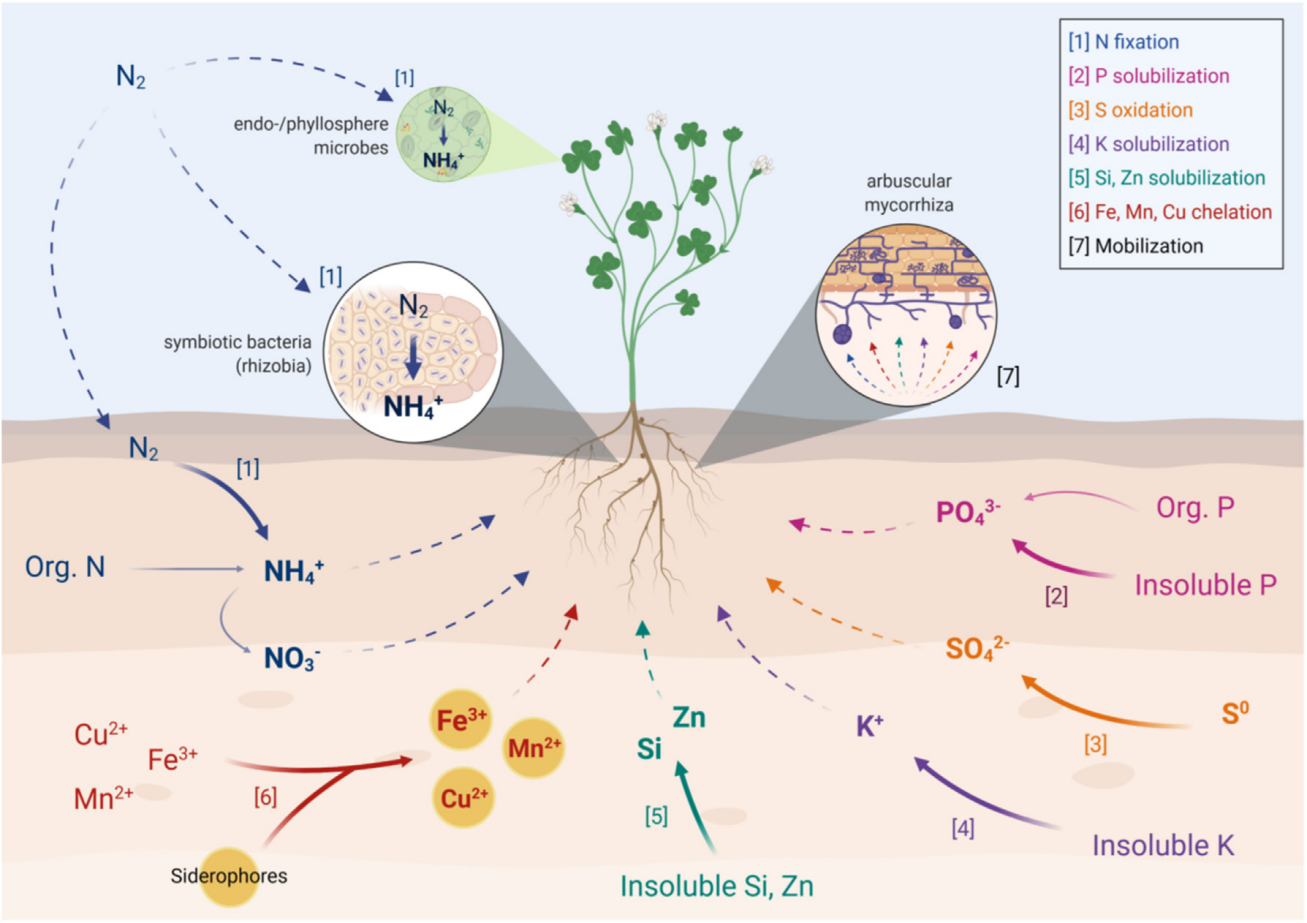
Key microbially-mediated nutrient transformation/acquisition pathways associated with biofertilizers. Full arrows represent microbial transformations whereas dashed arrows represent mobilization/movement of nutrients (Mitter et al., 2021).

Plants need 14 essential mineral elements to grow and develop, which are macronutrients – N, P, K, Ca, Mg and S, and micronutrients – Fe, B, Cl, Mn, Zn, Cu, Mo, and Ni (de Bang et al., 2021; Romera et al., 2021). Although most of the elements are found in soil, they cannot be taken up by the plants because they are in forms that plants cannot assimilate. Some of these elements are absorbed by plants only in certain forms like nitrogen that is absorbed as either nitrate or ammonia. As shown in Figure 1, using microorganisms will promote plant growth and provide plants with nutrients (Stamenković et al., 2018). Biofertilizers are classified based on the groups of microorganisms they contain and the functional features they have developed during the interactions with plants in the rhizosphere (Mącik et al., 2020; Raimi et al., 2017). Table 2 shows the general classification of biofertilizers – N-fixing, P-solubilizing, P-mobilizing, micronutrient solubilizer and plant-growth promoting rhizobacteria.

**Table 2:** Grouping of biofertilizers with functions (Umesha et al., 2018).

No.	Nature of organisms	Functions	Examples
1	Free-living	Nitrogen-fixing biofertilizers	*Azotobacter*, *Beijerinckia*, *Clostridium*, *Klebsiella*, *Anabaena*, and *Nostoc*
2	Symbiotic	Phosphorus-solubilizing biofertilizers	*Rhizobium*, *Frankia*, and *Anabaena azollae*
3	Associate symbiotic		*Azospirillum*
4	Bacteria		*Bacillus megaterium var phosphaticum*, *Bacillus subtilis*, *Bacillus circulans*, and *Pseudomonas striata*
5	Fungi	Phosphorus-mobilizing biofertilizers	*Penicillium* sp. and *Aspergillus awamori*
6	Arbuscular mycorrhiza		*Glomus* sp*., Gigaspora* sp*., Acaulospora* sp*., Scutellospora* sp*.*, and *Sclerocystis* sp*.*
7	Ectomycorrhiza		*Laccaria* sp*., Pisolithus* sp*.*, and *Boletus* sp*.*
8	Ericoid mycorrhizae		*Pezizella ericae*
9	Orchid mycorrhiza		*Rhizoctonia solani*
10	Silicate and zinc solubilizers	Biofertilizers for micronutrients	*Bacillus* sp*.*
11	*Pseudomonas*	Plant-growth-promoting rhizobacteria	*Pseudomonas fluorescens*

### 2.2 Liquid biofertilizers

Liquid biofertilizers are used as an alternative to carrier-based formulations (Mącik et al., 2020; Nagarajan, 2021). They are also called flowable or aqueous suspension. They are based on broth culture, mineral and organic oil, oil in water, and polymer based suspension (Bharti et al., 2017). Liquid biofertilizers typically consist of 10–40% microbes, 1–3% suspender ingredient, 1–5% dispersant, 3–8% surfactant, and 35–65% carrier liquid (water or oil) (Mishra and Arora, 2016). Liquid biofertilizers should contain special cell protectants that contribute to the development of cysts and dormant spores (Raimi et al., 2021).

Liquid biofertilizers are more attractive than solid inoculants because they have a long shelf life of 1.5–2 years, have no contamination, do not need sticky materials, can be used with modern machinery, can withstand high temperatures up to 45 °C, are easy to handle and use, are easy for adding ingredients that enhance the growth of microbial strains, and are easy to apply on both seeds and soil (Mahanty et al., 2017). Also, liquid inoculants have higher microbial densities that allow for lower dosages compared to solid-inoculants yet obtain the same effect (El-Ramady et al., 2018; Mącik et al., 2020).

The carrier material for liquid biofertilizers should be cheap, abundantly available, non-toxic, and easy to use. The carrier also must have a suitable pH, high water holding capacity, and physical and chemical homogeneity to enhance microbial growth (Raimi et al., 2021). Although liquid biofertilizers can be stored for a long time, microorganisms may face nutrient depletion, hypoxia, and environmental stresses which cause the microbial population to dramatically decrease. Therefore, special storing conditions are needed such as cool temperatures (Lee et al., 2016; Mącik et al., 2020).

Microbes in broth culture do not survive for a long time and lose their effectiveness on seeds (Mahanty et al., 2017). Hence, to let microbes survive for a longer time in liquid formulations, some additives are added such as sucrose, Arabic gum, glycerol, and polyvinylpyrrolidone, due to their ability to inactivate toxic compounds, improve adhesion to seeds, and improve microbial strains survival under different environmental conditions (Mącik et al., 2020). Liquid biofertilizers can reduce the use of chemical fertilizers by 15–40%. In addition, their dosages are less than solid biofertilizer by 10%, thus less amount is needed, allowing for smaller storage spaces (Riaz et al., 2020).

There are four types of liquid biofertilizers: suspension concentrates, ultralow volume suspension, oil-miscible flowable concentrate, and oil dispersion. Suspension concentrates are favored by farmers more than wettable powders because they are not dusty, easier to measure, and can be poured in spray tanks. Suspension concentrates are made by combining solid active ingredients with low water solubility and acceptable hydrolysis stability. Before using, suspension concentrates must be diluted in water. Using surfactants and other chemicals can increase their storage and solubility. Ultralow volume suspension is a ready-to-use formulation that can be dispersed by ultralow volume aerial or ground spray machinery in a very fine spray. Oil miscible flow concentrate is a dispersion of active ingredients in an organic liquid. It must be diluted before use (Mishra and Arora, 2016). Oil dispersion contains active ingredients in oil or in water immiscible solvent (Mącik et al., 2020). Oil is known to evaporate much less than water, so it stays in contact with plants for a longer time. It can be applied as an emulsion or an inverted emulsion (water in oil) (Mishra and Arora, 2016). Table 3 shows some of the companies that produce liquid biofertilizers in Jordan, India, Sri Lanka, and the United States.

**Table 3:** Companies providing liquid biofertilizers in Jordan, India, Sri Lanka and the United States.

Company	Biofertilizer	Composition	Possible function	Target crops	Reference
Al Anfal Fert. Industry CO., Jordan	Anfazyme	Seaweed with enzymes: 20–25% organic matter: vitamins, cytokinin, and auxins	Increases yield and improves plants quality	Fruits, vegetables, and field crops	(Al Anfal Fertilizer Industry Co., 2018)
Bio Power Lanka, Sri Lanka	Bio Vaccine	*Trichoderma viride*	High productivity, prevents the plants from diseases and rot	Used for nursery plants	(Bio Power Lanka, 2021c)
	Bio Gold	*Azotobacter chroococcum*, *Pseudomonas fluorescens*	Nitrogen fixing, phosphorus solubilizing, and prevent diseases	Fruits, vegetables, potted plants, and crop trees	(Bio Power Lanka, 2021a)
	Bio Phos	*Bacillus megatherium*	Phosphorus solubilizing	Plantation crops and vegetables	(Bio Power Lanka, 2021b)
Indogulf bioag, India	Fermogreen	*Nitrosomonadales*, *Rhizobiales*, *Cantharellales*, and 16 types of micro and micronutrients fortified with soil bacteria	Increases plant growth, fixes nitrogen, solubilizes phosphate like tricalcium iron and aluminum phosphate	Vegetables, fruits, medicinal crops, sugar crops, and plantation crops	(Indogulf Bioag, n.d-c)
	Revive (Bio.)	*Azotobacter chroococcum*, *Acetobacter aurantis*, *Pseudomonas striata*, *Paenibacillus mucilaginosus*, *Bacillus magaterium Var*. phosphate, *Trichoderma virens*, and *Streptomyces gelaticus*	Atmospheric nitrogen fixation, and solubilizes potassium and phosphorus salts	Aromatic crops, sugar crops, vegetables, fruits, and orchards	(Indogulf Bioag, n.d-d)
	Fermacto	Essential nutrients, nitrogen fixing bacteria, phosphorus fixing bacteria, cytokinin, betaines, and auxins	Reduces disease causing organisms and produces growth promoting hormones	Put on soil before sowing	(Indogulf Bioag, n.d-a)
	Multi-Bio	*Pantoea* spp., *azotobacter* sp., plant growth promoting rhizobacteria, LB planetarium, cyanobacteria, *Bacillus* sp*.*, and *Rhizobium* sp*.*	Provides plants with nitrogen, phosphorus, phytohormones, and iron	Tea, coffee, sugar, tomatoes, peanuts, lettuce, legumes, carrots, and rice	(Indogulf Bioag, n.d-b)
Agri life, India	AgriLife nitrofix	*Azotobacter chroococcum*, *Azotobacter vinelandii*, *Paenibacillus durus*, *Azospirillum Lipoferum*, *Rhizobium japonicum*, *Herbaspirillum frisingense*, and *Gluconocetobacter*	Nitrogen fixation	---	(Agrilife, 2020d)
	P Sol B	*Bacillus megaterium*, *Bacillus polymixa*, and *Pseudomonas striata*	Phosphorus solubilization	---	(Agrilife, 2020e)
	Mn Sol B	*Penicillium citrinum*	Mobilizes manganese	---	(Agrilife, 2020c)
	S Sol B	*Acidithiobacillus thioxidans*	Sulphur mobilizing	---	(Agrilife, 2020f)
	Zn Sol B	*Starkeya novella*	Zinc mobilizing	---	(Agrilife, 2020h)
	Fe Sol B	*Acidithiobacillus ferroxidans*	Iron mobilizing	---	(Agrilife, 2020a)
	Si Sol B	*Bacillus mycoides*	Solubilizes silica and helps the plant to tolerate biotic and abiotic stresses	Rice, sugarcane and cereal	(Agrilife, 2020g)
	K Sol B	*Frateuria aurantia* and *Bacillus mucilaginosus*	Potassium mobilization	---	(Agrilife, 2020b)
Nico Orgo, USA	BioAll	Nitrogen fixer, PSB, and potash mobilizing bacteria	Fixes atmospheric nitrogen, solubilize potash and phosphate	---	(Nico Orgo, 2015a)
	BioMicro	*Bacillus coagulans*, zinc, Sulphur, and ferrous mobilizing bacteria	Mobilizes iron, zinc, and Sulphur	---	(Nico Orgo, 2015b)
	K-Sol	*Bacillus coagulans*	Potash mobilizing	---	(Nico Orgo, 2015c)
	P-Sol	*Bacillus megaterium* and *Bacillus coagulans*	Phosphate solubilizing	---	(Nico Orgo, 2015d)
	N-Fix	*Azotobacter chroococcum* and *Azospirillum lipoferum*	Nitrogen fixation	---	(Nico Orgo, 2015e)
Novozymes, Denmark	Optimize Gold	*Sino rhizobium meliloti*, lipo-chitooligosaccharides (LCO)	Nitrogen fixation, and increases nutrient availability	Forages	(Novozymes, 2020f)
	Optimize	*Bradyrhizobium* sp*. arachis*, lipo-chitooligosaccharides (LCO)	Nitrogen fixation, helps with effective nodulation, and increase nutrient availability	Peanuts	(Novozymes, 2020a)
	TagTeam LCO	*Bradyrhizobium* sp*. arachis*, lipo-chitooligosaccharides (LCO), *Penicillium bilaiae*	Increases nitrogen fixation by forming nodules, and increases phosphate availability in soil which helps root and shoot growth as well as nitrogen fixation		(Novozymes, 2020c)
	Cell-Tech	*Rhizobium leguminosarum*	Nitrogen fixation, increases yield potential, and supports early vigor	Pulses	(Novozymes, 2020d)
	TagTeam LCO	*Rhizobium leguminosarum*, lipo-chitooligosaccharides (LCO), *Penicillium bilaiae*	Increases nitrogen fixation by forming nodules, and increases phosphate availability in soil which helps root and shoot growth as well as nitrogen fixation		(Novozymes, 2020b)
	Optimize XC	*Bradyrhizobium japonicum*, lipo-chitooligosaccharides (LCO)	Double the rate of early nodulation, nitrogen fixation, increases mycorrhizal association, and increases nutrient availability	Soybeans	(Novozymes, 2016b)
	TagTeam LCO XC	*Bradyrhizobium japonicum*, lipo-chitooligosaccharides (LCO), *Penicillium bilaiae*	Double the rate of early nodulation, nitrogen fixation, and increases mycorrhizal association		(Novozymes, 2016a)
	Cell-Tech	*Bradyrhizobium japonicum*	Nitrogen fixation, increases yield potential, and supports early vigor		(Novozymes, 2020e)

### 2.3 Mechanism of biofertilizers

Plant growth-promoting rhizobacteria stimulate plant growth through a variety of methods. They are frequently divided into direct and indirect methods. The bacteria may either directly increase plant growth by altering hormone levels or resource acquisition, or indirectly increase plant growth by reducing the impact of numerous pathogenic agents on plant growth and development (Daniel et al., 2022).

#### 2.3.1 Direct mechanism

Nitrogen becomes available to plants by the energy intensive process of biological nitrogen fixation (BNF) due to the fact that most of it is available in the atmosphere. Diazotrophs which consist of bacteria and archaea use the large stock of N_2_ from the soil to biologically fix nitrogen. Atmospheric N_2_ is catalyzed and reduced into ammonium (NH_4_^+^) by the *nifH* gene that encodes the highly conserved iron-protein subunit of the nitrogenase enzyme which consists of dinitrogenase reductase that has Fe as its co factor and dinitrogenase with Fe and Mo as its co factor as well as it being controlled by diazotrophs (Carpanez et al., 2022).

These microorganisms are divided into two groups symbiotic, and non-symbiotic. The symbiotic family consist of *Rhizobiaceae* and *Cyanobacterium anabaena* with azolla fern water where it has been used for more than 1000 years as a biofertilizer (Azolla-*Anabaena* symbiosis). The non-symbiotic family consists of *Azospirillum sp.* and *Cyanobacteria sp.* (Daniel et al., 2022; Mahmud et al., 2021). Phosphate is the second most important element for plant growth and development after nitrogen, except that it is not available to plants since it is in an insoluble form and only 0.1% is available to plants. Microorganisms help solubilize insoluble P like dicalcium phosphate, tricalcium phosphate, hydroxyapatite, and rock phosphate by acids and other mechanisms that reduce the soil’s pH into soluble forms of monobasic and dibasic which increase plants yield. Sometimes these bacteria mobilize the phosphorus (Mahanty et al., 2017; Riaz et al., 2020). Phosphate solubilizing bacteria efficiency depends on soil buffering capacity and its carbon and nitrogen sources (Raimi et al., 2017).

Potassium is also important for plant growth. Organic acids such as citric acid, formic acid, malic acid, and oxalic acid are produced by the dissolution of organic matter in soil. By providing protons and complexing Ca^+^ ions, these organic acids aid in the breakdown of K compounds. There are two main mechanisms in which Zn becomes available in soil. Those two mechanisms are dependent on pH. The first way is in acidic soils where Zn becomes available by cation exchange. The second mechanism is by forming zinc calcium carbonate by chemisorption (Mącik et al., 2020). With each unit increase in pH, Zn availability decreases by 100 times (Zeb et al., 2018). In an aerobic setting, iron is present as Fe^3+^. Because of this, microbes and plants are unable to absorb the majority of the iron. Typically, bacteria secrete siderophores, which are low-molecular-weight iron chelators with a strong affinity for complex iron, to get iron. Siderophores act as solubilizing agents for iron from minerals and organic molecules when there is a deficiency of iron. Several methods, such as chelation and release, direct uptake of siderophore Fe complexes, and ligand exchange events, are used by plants to absorb iron from bacterial siderophores (Daniel et al., 2022). Finally the last direct method is plant hormone production. Hormones like gibberellins, and acids such as acetic acid that enhances roots growth and uptake of nutrients by inhibiting some harmful and disease-causing microorganisms (Kumar et al., 2019) and indole-3-acetic acid (IAA) primarily controls plant cell differentiation, cell division, and root length (Mahmud et al., 2021).

#### 2.3.2 Indirect mechanism

Indirect plant growth stimulants include the production of hydrogen cynide (HCN) and ammonia. The generation of ammonia can help the host plant fulfill its needs for nitrogen while simultaneously lessening pathogen invasion. Because of its great toxicity against phytopathogens, HCN is extensively used as a biocontrol agent in agricultural settings. However, HCN is also used to chelate metal ions, and therefore indirectly contributes to phosphate availability. Another method for controlling plant infections has been thought to involve microbial production of chitinase. Chitinase causes the disintegration of the cell wall, which impairs the stability of the structure and prevents the growth of pathogens. An integral part of the fungal cell wall known as chitin (1,4-N-acetylglucosamine) is attacked by the enzyme chitinase. A defense mechanism employed by the host plant against a variety of plant diseases, induced systemic resistance (ISR) is brought on by jasmonate and ethylene signaling. ISR is recognized to lessen the severity of disease in a variety of plant species. Through the interaction of specific rhizobacteria with plant roots, it is possible to establish plant resistance against pathogenic bacteria, fungi, and viruses (Daniel et al., 2022; Mahmud et al., 2021).

### 2.4 Biofertilizer production

Biofertilizer was registered in the United Kingdom in 1896 and first produced by Nobbe and Hiltner as a product named “Nitragin” (Arora et al., 2020). It was also produced on a commercial scale in the United States, Malaysia and India in the 1930’s, 1940’s and 1960’s, respectively (Riaz et al., 2020). Biofertilizer production consists of six steps – screening for inoculant strains, deciding on biofertilizer functional traits, product formulation, strain cultivation, testing product type and efficiency, and commercial production (Raimi et al., 2021). Each of these steps is crucial to achieving a high-quality biofertilizer and must be carried out under strict conditions (Mącik et al., 2020).

**Figure 2:**
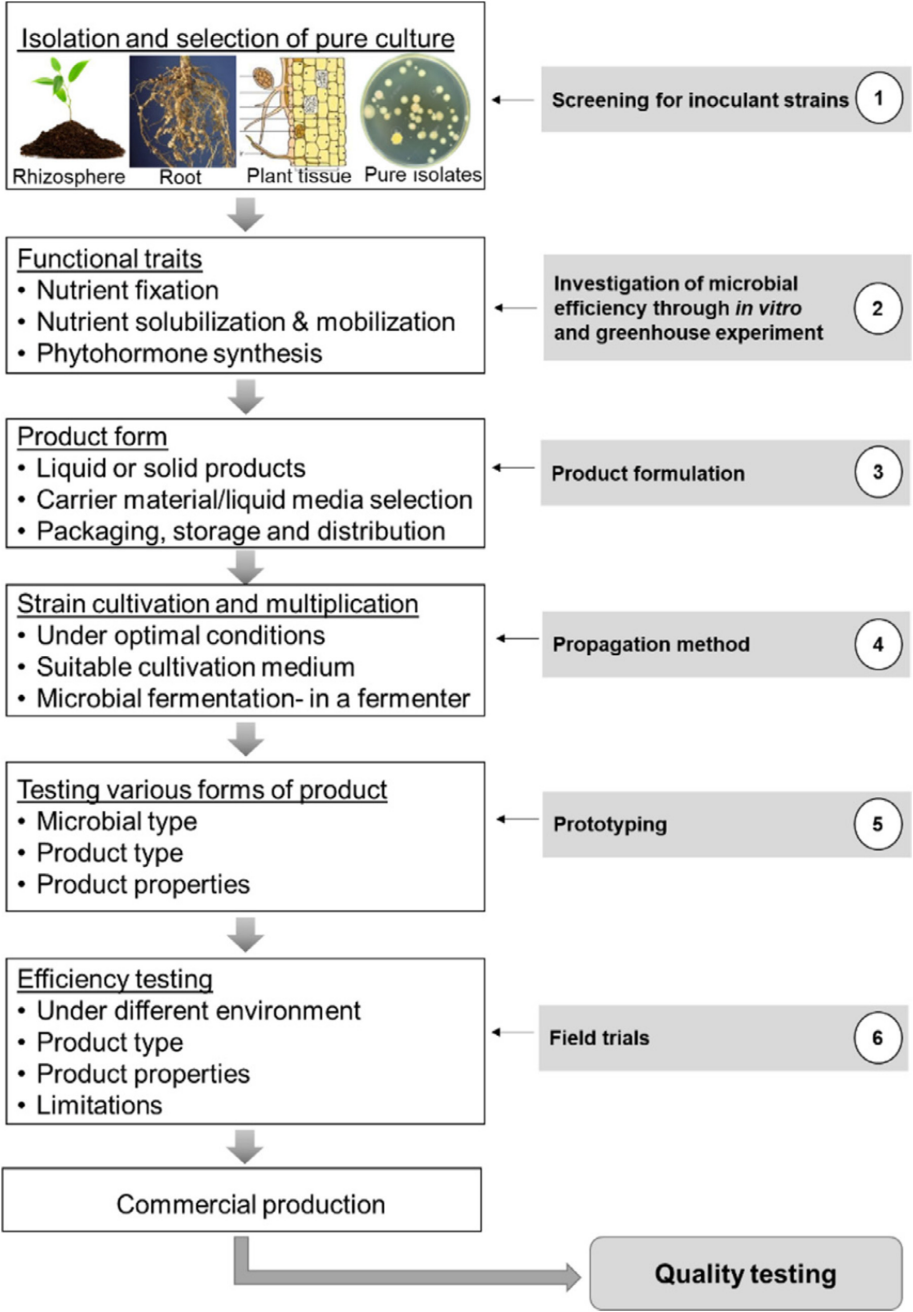
Standardization process for commercial biofertilizer production (Reprinted from Raimi et al. (2021), with permission from Elsevier).

As shown in Figure 2, in the first step, the microbial strains are isolated from soil, rhizosphere, and plant tissues such as stems, leaves, seeds, and roots. These strains must withstand the different cultivation methods. Then the microbes that can help enhance the plant growth are identified, isolated, and the functional traits are decided. Culturing improvements broaden the range of recovered microorganisms, increase the chances of discovering useful characteristics (El-Ramady et al., 2018; Kaminsky et al., 2019; Raimi et al., 2021).

In the second step, a pure culture is selected based on the desired functional traits like nitrogen fixation, nutrient mobilization and solubilization, and phytohormone production. The suitability and applicability of the selected strains are tested using different lab testing methods, for example, growth on selective media and quantitative testing to determine efficacy degree (Raimi et al., 2021). Also, before producing biofertilizers on a commercial scale, biofertilizer must undergo greenhouse tests to determine their efficiency before application on fields to make sure that it does not have any eco-toxicological effects and has beneficial impacts in promoting plant growth (Mącik et al., 2020). Moreover, the strains are analyzed to see how they promote plant growth. This knowledge can help in formulating the best formula for biofertilizer that can work in different ecosystems, since it is not profitable to produce a biofertilizer for each soil type.

In the third step, the carrier material, which is an inert material that transform microorganisms from the biofertilizer to the soil, is selected, either carrier-based or liquid (broth, broth + polyvinylpyrrolidone). Choosing the suitable carrier is very important to keep the microbes alive and in the right amount must be non-toxic, biodegradable, environmentally friendly, stable at room temperature, and cheap. Compared to solid carriers, liquid inoculants do not need sticky materials, need a fewer number of cells, and can be put in a bottle in larger amounts (El-Ramady et al., 2018; Raimi et al., 2021).

In the fourth step, the microbial strains are cultivated and multiplied using fermenters in laboratories under optimal conditions, as well as the appropriate reproduction method. Solid state and liquid fermentation methods are used for producing biofertilizers. In the fifth step, different types of the product (microbial type, product type, and product properties) are tested to select the one with the best performance. In the sixth step, large-scale field testing of biofertilizers is done to determine their efficiency and shortcomings in a variety of ecological regions and environments before finalizing a standardized method for production and processing (Raimi et al., 2021). Finally, the produced biofertilizers must be packed and the package must contain the following information: product name, date of production in addition to expiry date, the microbial strains in it, target crops, name and address of manufacturer, and instructions and recommendations for application (Mącik et al., 2020).

## 3 Liquid inoculants

For liquid biofertilizers, peat is the most used carrier for biofertilizers due to its properties in supporting the microorganism’s growth and survival. Still, due to its high sterilization cost, difficulty of application in large fields, and difficulty in processing, liquid inoculants were invented to be used as an alternative to solid inoculants. Liquid inoculants can be made from single mixed cultures that improve the cell survival in application and storage conditions (Buntić et al., 2019). The selected carrier should provide the microorganisms with a protective and suitable medium to survive, as well as staying effective over a long period of release. They also must be at neutral or near neutral pH in addition to being environmentally friendly (Buntić et al., 2019; Zommere and Nikolajeva, 2017).

Kumar et al. (2019) tested the survivability, nitrogenase activity, indole acetic acid production, ammonia, and siderophore production of 43 isolates obtained on Jensen’s medium of *Triticum aestivum* (wheat), *Zea mays* (Maize), *Solanum tuberosum* (potato), *Aloe baradensis* (*Aloe vera*), and *Bacopa monnieri* (Brahmi) soil samples*.* Four different liquid carriers of compost tea, biogas slurry, vermiwash, and minimal growth medium (peptone water) were used to develop the liquid biofertilizer. The viable cell count was counted over six months with samples drawn at monthly intervals. All the 43 bacterial strains were compared to a reference strain of *A. chroococcum*. It was found that only 18 isolates produced more than 150 nmol C_2_H_4_ h^−1^mg^−1^ of protein nitrogenase activity. In addition, out of these 18 isolates, only six showed higher nitrogenase activity compared to the reference bacterial strain of *A. chroococcum*, 13 produced IAA, nine produces siderophores, and 11 produced ammonia. Out of these isolates, the isolate from wheat rhizosphere was the most efficient. Based on the 16S rRNA gene sequencing, it was identified as *S. rhizophila* (WT-A2). As for the survivability test, only the most efficient isolate of WT-A2 was tested. The compost tea carrier showed a mean value of cell count higher than that of the reference strain. After compost tea, biogas slurry was the second-best carrier followed by vermiwash and minimal growth medium. Glycerol can also be added to liquid carriers to increase biofertilizer’s shelf life.

Gopi et al. (2019) developed an optimized liquid formulation of PGPR mix-I containing component cultures – *Azotobacter chroococcum*, *Azospirillum lipoferum*, *Bacillus megaterium*, and *Bacillus sporothermodurans*. This study was done using totally randomized design of liquid media. The liquid media used were 2% glycerol, 2% polyvinylpyrrolidone (PVP), 15 mM trehalose, 1% glycerol +1% PVP, 2% glycerol, 1% trehalose, 1% yeast extract, 1% PVP, and 1% proline, with a talc based formulation as the standard. All of the inoculated flasks were kept for 18 months at room temperature in a rotary shaker with a constant speed of 100 rpm and were grown on a medium of 1:1:1:1 of Jensen’s broth, nutrient agar, Pikovaskaya’s broth, and nitrogen-free bromothymol blue (NFB). Samples were drawn at bimonthly regular interval. After the first two months 2% glycerol medium showed the highest number of bacterial cells in all of the strains, while at the end of the study 15mM trehalose had the highest viable count due to the protective capability of trehalose in enhancing cell tolerance to desiccation, osmotic pressure, and temperature stress.

Elsakhawy et al. (2021) tested the survivability of *Rhizobium* sp. SARS 81 maintained on a yeast extract mannitol agar medium (YEMA) and used several additives over 12 months. The bacterial strain was tested on several compositions of YEMA with different compositions of acetone, glycerol, Gum Arabic, and PVP. The cell was counted on a monthly basis. It reached its maximum after the first two months. As for some additives like PVP and PVP + glycerol, they showed a sharp decline after the first six months and reached lower count at the end of the six-month period. Yet, the compositions that contained acetone survived for a longer time. The treatment with PVP + acetone reached a count of 9.93 log CFU mL^−1^ at the end of the storage period.

Lee et al. (2016) tested the surviavability of *Rhodopseudomonas palustris* strain PS3 on six additives of alginate, polyethylene glycol, polyvinylpyrrolidone-40, glycerol, glucose, and horticultural oil under low (4 °C ± 2), medium (25 °C ± 2), and high (40 °C ± 3) temperatues. The PS3 strain was grown on purple non-sulfur bacteria (PNSB) broth, incubated for 22 h at 37 °C with 200 rpm until the cell count reached 2 × 10^9^ CFU mL^−1^. The cell count was adjusted at 9 log CFU mL^−1^ for all of the six additives. All additives were tested at different concentrations (%*w/v*). under the low temperature conditions, 0.5–5% of horticultural oil, 3% glucose, 3% glycerol, and 5% PVP had the highest viable cell count of PS3 > 8 log CFU mL^−1^. At medium temperatures, 1% glucose, 0.5% alginate, 2% PVP, and 0.5 and 1% horticultural oil supported the growth of 5.9–6.5 log CFU mL^−1^ of PS3. Meanwhile, at high temperatures, 1% glycerol, 0.5 and 2% horticultural oil, 3% glucose, and 0.5% PEG supported the growth of 4.7–5.3 log CFU mL^−1^ of PS3. As seen from the study, only 0.5% horticultural oil supported the growth of PS3 under low, medium and high temperature conditions compared to the control treatment.

Karadbhajne and Jambhekar (2018) tested five different bacterial strains survival over one and a half years to see their viable cell count. This study was done on a pilot scale using a cheap liquid media and showed great results of the effectiveness of liquid inoculant with high viable cell count. All samples were grown on nutrient agar media for 48 h at 30 °C. Samples were drawn at regular interval of 30 days over one and a half year. *Acetobacter* (nitrogen fixing), *Azotobacter* (nitrogen fixing), *Frateuria* sp*.* (potassium mobilizing), and *Pseudomonas* sp*.* (phosphate mobilizing) had 10^14^–10^15^ CFU mL^−1^ viable microorganisms at 28 °C, whilst *Trichoderma* (plant growth promoting bacteria) had less than 10^8^ CFU mL^−1^. Another experiment was done using *Rhizobium leguminosarum* bacteria on nutrient agar broth with additives of polyvinylpyrrolidone at varying concentrations of 2%, 2.5%, and 5%. The concentration at 2.5 % showed the longest survival time of over 720 days with 9.903 CFUmL^−1^ (Trivedi et al., 2016). Also, bacteria grown on acetone additives (10 mg L^-1^) had a shelf life up to 12 months at room temperature. These experiments showed that a shelf life up to two years is achievable with liquid biofertilizers.

Liquid biofertilizers can be made from the by-products and wastes from several industries. Table 4 shows the possible liquid media sources derived from wastes – the by-product of monosodium glutamate (MSG), fruit and vegetable, glycerin pitch from oleochemical industries, cane molasses, corn steep liquor, and wastewaters.

**Table 4:** Summary of studies using wastes as growth media for bacteria.

Bacterial strain	Liquid media sources	Viable cell count (CFUmL^−1^)	Spore count (CFUmL^−1^)	References
*Bacillus* sp*.* and other bacteria	MSG by-product	7.45 × 10^9^	6.45 × 10^6^	(Namfon et al., 2017)
*Bacillus siamensis*	Anaerobic digestate of fruit and vegetable waste (with sucrose additive from sugar beet)	1.5 × 10^8^	-	(Pastor-Bueis et al., 2017)
*Lactobacillus*	Glycerin pitch	-	-	(Nasarudin et al., 2020)
*Bacillus subtilis*	Corn steep liquor	3.9 × 10^9^	-	(Z. He et al., 2020)
*Azotobacter*	Cane molasses	1 × 10^8^	-	(Hindersah et al., 2020)
*Bacillus*		-	1 × 10^10^	
Microbial consortia	5 isolates from activated dairy sludge, and 1 isolate from marine coastal waters at 1:1 ratio	7.36 × 10^7^	-	(Halder et al., 2020)
*Bacillus anthracis, B. fusiformis, and B. licheniformis*	Mixed fishery wastewater from mackerel and brown seaweed	16.2 × 10^7^	-	(Jung and Kim, 2020)

MSG by-products are difficult to treat but they are rich in nutrients and available at low cost. Hence, they can be used as a substrate for liquid biofertilizers. In Namfon et al. (2017), MSG by-product was used as the main carbon and nitrogen source for the cultivation of bacteria. The study concluded that the best conditions for producing liquid biofertilizer from MSG by-products at economical scale were at a reducing sugar of 20 g L^−1^ and an inoculum level of 10% for higher viable cell count. The spore count was however low which is an aspect for improvement to achieve longer shelf lives.

Glycerin pitch is a waste product produced from oleochemical industries and possesses risk to the environment when disposed. Glycerin pitch consists mainly of glycerol >55% that can be used as a carbon and energy source for fermentation processes. In Nasarudin et al. (2020), three samples were prepared with ratios of 1:1, 1; 2, 2:1 of glycerin pitch to *Lactobacillus*. After mixing them, they were left for 14 days before using it on cucumber seeds. The number of L*actobacillus* bacteria was determined based on the increment of the turbidity (in nephelometric turbidity units, NTU) in addition to the increase in dry mass concentration in the prepared samples while its size was determined in the order of wavelengths of the visible light. Turbidity increased from 300 NTU to about 380 NTU in 200 h at the glycerin pitch to a *Lactobacillus* ratio of 2:1, indicating a consistency in the growth of *Lactobacillus*. The pH in the three samples was reduced. Fumaric acid, lactate and acetate were formed by *Lactobacillus* that exhibited nutrient-solubilizing characteristics, which helped to tackle hard-to-dissolve nutrients and made them available to plants. Carbon content was also reduced by *Lactobacillus* bacteria. The reduction in carbon indicates that it was converted into simple and useful forms to the plant. Another parameter was an increase in the liquid viscosity from 2.35 to 3.75 MPS. This viscosity is related to the increase in microbial density which enables longer shelf lives.

In Pastor-Bueis et al. (2017), the anaerobic digestate from the vegetable and fruit industry was used as the main ingredient of the growth medium for PGPR *Bacillus siamensis*, which was isolated from the rhizosphere of a sweet pepper crop. The field tests showed that treatments inoculated with the biofertilizer made of anaerobic digestate at 50% dilution and 2.3% sugar beet molasses and fertilized with decreased mineral N at 80% produced much better results than the ones with merely 80% of N and 100% of N without the biofertilizer.

High-cost materials are usually used for the fermentation of *Bacillus subtilis* such as corn starch and peptone. Due to this fact, there is a need to find cheaper and more readily available alternatives. Corn steep liquor (CSL) is a concentrated organic wastewater that is produced from corn wet milling. It is rich in amino acids and protein. In He et al. (2020), it was found that, as the concentration of CSL increased, it inhibited the growth of *Bacillus* bacteria. Therefore, the best concentration was between 100 to 200 g L^-1^. Similarly, organic wastes from agricultural activities such as sugar cane byproduct and cane molasses can be used as a cheap liquid media to produce liquid biofertilizers. Cane molasses is rich in sugar (46–52%) which is an energy and carbon source for *Azotobacter* and *Bacillus*. It is also rich in acids, as well as calcium, potassium, sulfate, sulfite, chloride, sodium, and magnesium which are important for the growth of the bacteria. In Hindersah et al. (2020), two types of *Bacillus* (*B. magaterium* and *B. subtilis*) and *Azotobacter* (*A. chroococcum* and *A. vinelandii*) were used and kept on nutrient agar and Ashby’s mannitol agar. Both *Azotobacter* and *Bacillus* species produced phytohormone IAA, exopolysaccharide, cytokinin (Cks) and gibberellins (GAs). In this experiment, molasses was the only carbon source while ammonium chloride was added as a nitrogen source. The 1:1:1:1 composition gave the best result over one month of storage time where the viable cell count did not differ significantly. The presence of IAA, Cks, GAs, and zeatin indicates that the waste product of sugar cane industries is a good source for producing liquid biofertilizers.

Dairy wastewater and mixed mackerel and brown seaweed wastewater were also used as the liquid media sources as they are nutritionally rich (Halder et al., 2020; Jung and Kim, 2020). In Halder et al. (2020), dairy wastewater was converted into biofertilizer and tested on mung bean in India. Four treatment sets were compared – raw effluent, microbially treated effluent, tap water with chemical fertilizer and tap water (as the control). Microbially treated dairy effluent yielded the best results in terms of percentage survival, nodulation, and seed yield, indicating that the treated effluent can be fully used for irrigation, resulting in zero liquid discharge, thus increasing the economic benefits of dairy industries. Similarly in Jung and Kim (2020), mixed fishery wastewater from mackerel and brown seaweed was converted into biofertilizer using *Bacillus* species. The 72-hour culture broth yielded a biofertilizer which met the standard content of N, P, K, heavy metals and the number of viable cells. When applied to one-month-old lettuce plants, an enhanced growth rate was observed compared with controls, with high chlorophyll and carotenoid content, high antioxidant activity and no permeation of pathogens.

## 4 Effects of liquid biofertilizers on plant growth

Table 5 summarizes some selected studies conducted on different crops with different liquid inoculants and bacteria strains. Buntić et al. (2019) did an experiment on alfalfa seed, which is a leguminous plant of the pea family (Fabaceae) used to fix nitrogen to other plants, because it houses rhizobia bacteria. Alfalfa is planted as a cover crop to enhance the soil properties and increase its nutrient levels. It can also tolerate drought, heat, and cold weather. The *Sinorhizobium meliloti* L3Si strain was grown in yeast mannitol broth (YMB). As for the liquid inoculant, ten different media were prepared. They were added in combination or separately. For the survival of L3Si during a storage time of 150 days, the most suitable liquid inoculant was found to be a glycerol medium formulated with agar or sodium-alginate. *Sinorhizobium meliloti* L3Si’s effectiveness on alfalfa seeds were studied on nodulation, plant height, shoot dry weight, and nitrogen content in the shoot dry weight. After one-month storage, alfalfa seeds pre-inoculated with YMB, YMB with agar, and YMB with sodium-alginate for up to three months produced successful alfalfa plants with nitrogen content ranging from 3.72 to 4.19%.

**Table 5:** Selected studies conducted on different crops with different liquid inoculants and bacteria strains in recent years.

Plant	Liquid Inoculant Medium	Bactria type	Cell count (CFU mL^−1^)	Nitrogen (%, unless specified)	Phosphorus (%, unless specified)	Potassium (%, unless specified)	Nodulation (%)	Plant growth (cm/plant)	Root dry mass (g/plant)	Shoot dry weight (g/plant)	References
Alfalfa	Yeast mannitol broth agar	*Sinorhizobium meliloti* L3Si	8.1✕10^8^	3	-	-	100	16	-	0.018	(Buntić et al., 2019)
*Vitis vinifera* L. (grape)	Nutrient agar	*Pseudomonas putida* Rs-198	1.4✕10^13^	44 mg kg^−1^	29 mg kg^−1^	192 mg kg^−1^	-	-	-	-	(Lu et al., 2020)
Cucumber (*Cucumis sativus)*	Glycerin pitch	*Lactobacillus*	-	-	-	-	-	40	-	-	(Nasarudin et al., 2020)
Pepper (Capsicum spp.)	Nutrient medium	*Pseudomonas putida* Rs-198	1✕10^9^	18	25	25	-	-	0.434	3	(Y. He et al., 2019)
*Oryza sativa* L. (rice) Toledo	N/A	*Azospirillum brasiliense* Ab-V5 and Ab-v6	2✕10^8^	-	-	-	-	108	32	195	(Guimarães et al., 2020)
*Oryza sativa* L. (rice) Palotina								106	32	189	
*Oryza sativa* L. (rice) Cascavel								111	33	192	
*Oryza sativa* L. (rice) São Miguel do Iguaçu								107	33	189	
*Green gram (Vigna radiata L)*	Tap water	*Chlorella vulgaris* at 100% concentration	-	25∗	43	17∗	133 (on day 6)	42	0.68	0.7	(Dineshkumar et al., 2020)
		*Spirulina platensis* at 100% concentration		46	66	17∗	142 (on day 6)	40	0.67	0.66	
*Cotton*	Nutrient agar	*Pseudomonas putida* Rs-198	1✕10^14^	-	-	-	-	20	0.19	-	(Y. He et al., 2016)
*Onion*	Nutrient broth	*Azotobacter* sp*. + Sphingobacterium* sp*. + Burkholderia* sp*.*	1✕10^10^	-	-	-	-	42	-	-	(Tinna et al., 2020)
*Chickpea*	-	*Bacillus subtilis*	3.6✕10^9^	30	-	18	-	-	2	3.0	(Abd_Allah et al., 2018)
*Wheat*	-	*Azotobacter* + PSB + KMB + ZSB	>8.5 ✕10^8^	-	-	-	-	92	-	-	(Jain et al., 2021)

Lu et al. (2020) did an experiment on *Vitis vinifera* L. (grapes) by preparing four different concentrations of *P. putida* Rs-198 in a nutrient agar liquid medium. The most effective number of Rs-198 was 1.44 × 10^13^ CFU mL^−1^ in the sample, called BFP3 in terms of soil physiochemical properties, appearance, and quality of grape. The use of Rs-198 increased the growth and quality of grapes as it increased the grape width by 4.4%, length by 6.2%, and weight by 17.2% compared to non-inoculated grapes. The physiochemical properties and fertility of soil was increased significantly by the application of Rs-198 due to the increased enzymes’ activity. The alkaline phosphate and invertase activities were increased by 27.15% and 48.33%, respectively, and the urease was almost the same. Another experiment was done on pepper seeds by He et al. (2019) where they compared inoculated and non-inoculated seeds. The inoculated plants had higher contents of chlorophyll a, chlorophyll b, and carotenoids, with 162%, 152%, 84%, 108%, 97%, and 131% more flowers, leaves, root fresh weight, shoot fresh weight, root dry weight, and shoot dry weight, respectively. The contents of N, P, K and Na in the stems and leaves were increased because Rs-198 induced the alteration of Na uptake by modifying the expression of Na^+^/H^+^ antiporter.

In Nasarudin et al. (2020), three samples were prepared using different ratios of glycerin pitch and *Lactobacillus* bacteria. Glycerin pith was used successfully as a growth medium and carbon source for *Lactobacillus* bacteria. When applied to cucumber, its height was almost doubled when compared to commercial fertilizers. Guimarães et al. (2020) did an experiment on *Oryza sativa* L. (rice) at different locations in Brazil that had different soil types to study the efficiency of the *Azospirillum brasiliense* liquid and solid biofertilizers. Seven samples were prepared using commercial liquid and solid biofertilizers. The sample with 40 kg N ha^−1^ of Nitro 1000 liquid biofertilizer containing 2 × 10^8^ CFU mL^−1^ *Azospirillum brasiliense* showed the best results in the four different regions of Toledo, Palotina, Cascavel, and São Miguel do Iguaçu. This liquid biofertilizer sample also showed better performance and efficiency than that using solid inoculant.

Microalgae-based biofertilizers have also been evaluated. In Dineshkumar et al. (2020), liquid biofertilizers extracted from *Chlorella vulgaris* and *Spirulina platensis* were applied on green gram *Vigna radiata* (L.). Among all the treatment concentrations, the two treatments at 100% showed the highest yield of green gram yielding 29 and 30 pods plant^−1^ for *Chlorella vulgaris* and *Spirulina platensis,* respectively. Also, the plant height, root and shoot dry weight, nutrient compositions were increased significantly. Amino acids were found to be higher in plants treated with *Chlorella vulgaris,* whereas N, P, and K were found to be higher in plants treated with *Spirulina platensis* (Dineshkumar et al., 2020)*.*

He et al. (2016) experimented with the effect of cotton plant growth treated by free and encapsulated *Pseudomonas putida* Rs-198 under salt stress. The encapsulated *Pseudomonas putida* Rs-198 with 2% salt stress condition did not increase the plant height but the root length, fresh and dry weights. While the encapsulated *Pseudomonas putida* Rs-198 without salt stress enhanced the plant height, fresh and dry weight. It can be concluded that the encapsulation of bacteria with alginate can enhance plant growth in nutrient deficient soils. Also, Abd_Allah et al. (2018) tested the adaptive mechanism of chickpea plants treated by *Bacillus subtilis* under saline conditions. The treated plants with *Bacillus subtilis* under saline conditions reduced sodium accumulation and enhanced nitrogen, potassium, calcium, and magnesium contents in plants, as well as enhancing the membrane stability under salinity and non-salinity conditions.

Jain et al. (2021) experimented with the effects of bacterial consortia consisting of *Azotobacter*, phosphorus solubilizing bacteria, potassium mobilizing bacteria, and zinc solubilizing bacteria on the growth of wheat in two successive *rabi* seasons of 2017–2018 and 2018–2019. Two microbial consortia where prepared – one of them contained all the mentioned bacterial strains above (BF_2_) and the other with the absence of ZSB (BF_1_). The best results were obtained from the treatment combination of using BF_2_ in addition to the inorganic chemical fertilizer at 100% recommended doses of NPK (120 kg N + 60 kg P + 40 kg K), where it enhanced plant growth, grain yield, and benefit-cost ratio. Statistically, BF_1_ and BF_2_ with 75% of the recommended dose of inorganic fertilizer can be used, yielding very close results to BF_2_ + 100%, so it could save about 25% of inorganic chemical fertilizer. In a similar manner, Tinna et al. (2020) experimented with a combination of a bacterial consortia with an inorganic chemical fertilizer. They reached the same conclusion that a combination of both chemical and biofertilizer could save up to 25% of chemical fertilizer, enhancing the plant height, bulb yield, and number of leaves of onions.

## 5 Challenges and future perspectives

Most of the biofertilizers produced are effective on certain crops, soil types, and climates. Plant growth and development is affected by several biotic and abiotic stresses in the soil environment (Bramhachari et al., 2018). Moisture is needed for nutrient uptake and absorption by plants. Yet, due to climate change, drought stress is considered one of the most serious abiotic environmental stresses affecting the hemostasis of soil, and the morphological, physiological, and nutritional traits of plants (Anli et al., 2020; Borowik and Wyszkowska, 2016). The results of applying liquid biofertilizers are sometimes unpredictable like what happened in Belgium in the 1999–2000 season when a biofertilizer had positive effects at the start of the winter season, but as weather conditions worsened, the final yield did not reach its full potential (Stamenković et al., 2018). The same happened in Uruguay, where at first plant growth was observed, but decreased with time with no significant difference between inoculated and non-inoculated plants. When the experiment was done outside of the growth season, the results were negligible (Dobbelaere et al., 2001). Therefore, biofertilizers must be developed to resist drought stress and other environmental stresses due to seasonal changes, as well as to search for microbial strains that can withstand stressful conditions. By doing so, farmers in harsh environments or developing countries will benefit from such biofertilizers (Santos et al., 2019).

From the literature review presented here, it is apparent that there is a lack of field tests to test the efficiency of biofertilizers. The effect of *Bradyrhizobial* bacteria on peanut production in Argentina was studied at four different locations to test the inoculation efficiency. The results showed that there was a response only at one location because the soil was N-deficient (Valetti et al., 2016), suggesting that biofertilizer efficiency varies through different soil types in different ecological regions. Hence, more field trials to test biofertilizer efficacy and limitations are essential on a case-by-case basis to better understand the relationships among crops, nutrients, and microorganisms present in the soil, keeping in mind that biofertilizers are not a source of nutrients (Raimi et al., 2021).

When transforming liquid biofertilizer from laboratory scale to large scale, it may act poorly due to other variables not being studied on the lab scale. These variables must be taken into consideration to produce liquid biofertilizers suitable for various weather conditions and soil types. Another challenge is that for liquid biofertilizers, different or more advanced machinery might be required for large-scale application, thus making it energy intensive. Therefore, liquid biofertilizers must be produced in a way that can be used on the current or low-cost machinery.

Since a viable cell count is the most important factor in evaluating the quality of liquid biofertilizers, there is a need to develop new technologies to target the additives and carriers that are cheap, easy to use, and effective in supporting a large number of viable cells count with over 1 × 10^8^ CFU mL^−1^ over long periods of time for transportation and storage (Hindersah et al., 2020; Mitter et al., 2021). Waste by-products that are rich in valuable sources such as nutrients, chemical oxygen demand, sulfates, chlorine, ammonia, and other non-toxic, environmentally friendly substances, can be used as an alternative source for biofertilizer carriers because some industries have difficulties in treating their wastes and wastewater or have to pay to dispose of them (Arumugam et al., 2021) (Arumugam et al., 2021; Namfon et al., 2017; Nasarudin et al., 2020; Rai et al., 2022). This would be a step towards circular economy where wastes or wastewaters can be applied as the liquid media to produce liquid biofertilizers. More research is needed in this area to explore which potential by-products that are rich in beneficial substances to support the growth of bacteria for producing biofertilizers that are cost effective while ensuring the safety of workers and the environment in the long term.

To evaluate their viability, a cost-benefit analysis must be done to deduce the profitability of biofertilizer. After discounting the gross cost and benefit, an organization will be profitable if the benefit-to-cost ratio surpasses 1. For example, the benefit-cost ratio was found to be 17 for soybean by fixing 100 kg N ha^−1^, and 416 for clover by fixing 200 kg kg N ha^−1^ based on n-fixation since they are legume crops (Mulongoy et al., 1992). Also, the biofertilizer energy requirement is fully paid by nature when compared to chemical fertilizers that require 80 MJ for N, 12 MJ for P, and 8 MJ for K leaving many small-holder farmers unable to afford the expensive energy bill. In legume plants, 48–300 kg N ha^−1^ is fixed in a season. The amount of biofertilizers required to supply plants with the same amount of nutrients is much less than chemical fertilizers. Additionally, this low-cost method of supplying nutrients to soil makes it attractive to small-holder farmers (Raimi et al., 2017). Therefore, more studies are required to conduct cost-benefit analyses of biofertilizers based on plant yields.

Some biological molecules such as flavonoids, strigolactones, and polysaccharides can promote a symbiotic relationship between arbuscular mycorrhiza fungi and the host plant, as well as rhizobium during nodule formation. For improved product formulation, these compounds should be explored (Raimi et al., 2021). The existing bacterial strains help in improving one trait of the plant, but scientists may need to develop genetically engineered strains that are more efficient while ensuring that these developed strains do not cause any hazards or risk (Mahanty et al., 2017; Mitter et al., 2021).

Some studies have shown that biofertilizers can affect the surrounding environment by introducing microorganisms that can affect the structure of native microflora and some non-target effects like changes in biogeochemical cycles, soil texture, and soil properties. However, it is still unknown how these microorganisms react with the presented microflora. In addition, the severity of the changes on the ecological systems have yet to be revealed. No recent studies have explored the safety of bioinoculants for commercial use (Mahanty et al., 2017).

In addition, there is a need to improve education about biofertilizers and their long-term benefits compared with chemical fertilizers, and to correct the misconception about microorganisms being a source of disease. Biofertilizers will require new and innovative techniques for the growth, transportation, formulation, storage, and application of microorganisms as they go from small scale (laboratory and greenhouse tests) to large scale production. More investment is needed in the existing and future technologies via research to produce cost-effective and environmentally friendly biofertilizers. However, there is a lack of communication between farmers, industry, researchers, and governmental sectors. Multi-stakeholder partnerships are definitely crucial in order to develop biofertilizers that do the best job possible.

## 6 Conclusion

Liquid biofertilizers consist of living microorganisms that enhance soil properties and increase plant growth and yield. Liquid biofertilizers have been applied to different crops and yielded the best results when compared to other types of chemical or carrier-based fertilizers. In some cases, plant growth increased two-fold. Biofertilizers can be produced by using a single or a mix of microorganisms based on the role the biofertilizer is produced for. Also, liquid biofertilizers can be made from wastes and by-products of some industries as they could be a suitable and low-cost option for the growth of the bacterial cells instead of using specially made media. Finally, in order to develop effective liquid biofertilizers, more research is needed to overcome their limitations in the aspects of better climate adaptation, longer shelf life, better liquid inoculant, and use of low-cost or existing machinery for large-scale application. Cost-benefit analyses, field trials, long-term safety and effectiveness evaluations, and multi-stakeholder partnerships are the essential elements to evaluate the feasibility of the liquid biofertilizer on a case-by-case basis, taking into consideration the location, crop type, soil type, and climate.

## Declarations

### Author contribution statement

All authors listed have significantly contributed to the development and the writing of this article.

### Funding statement

This research did not receive any specific grant from funding agencies in the public, commercial, or not-for-profit sectors.

### Data availability statement

Data will be made available on request.

### Declaration of interest’s statement

The authors declare no competing interests.

### Additional information

No additional information is available for this paper.
